# Embodied earth kinship: interoceptive awareness and relational attachment personal factors predict nature connectedness in a structural model of nature connection

**DOI:** 10.3389/fpsyg.2024.1400655

**Published:** 2024-08-29

**Authors:** Lindsay Branham

**Affiliations:** Department of Psychology, University of Cambridge, Cambridge, United Kingdom

**Keywords:** nature connection, body awareness, relational attachment, interoceptive awareness, climate change, social and ecological justice, connectedness, climate resilience

## Abstract

Previous research has found that nature connectedness, an experiential close connection to nature with cognitive, affective and physical benefits, profoundly impacts individual wellbeing and subsequently increases pro-environmental behaviors. However, little is known about the personal and contextual factors that predict nature connectedness. Testing theory derived from a qualitative interpretative phenomenological analysis study, this research addresses the lacuna in the literature. A structural equation model analysis finds that interoceptive awareness significantly predicts nature connection, that secure attachment to nature significantly explains this relationship, and that these inter-related constructs predict both pro-environmental behavior and wellbeing. This revised model of nature connection indicates important antecedents for the human-nature bond, illuminating in particular that the interpersonal relational processes foundational for close bonding with humans also occur in bonding with nature. Structural equation modeling indicates that emotional awareness is the dimension of interoceptive awareness that most significantly predicts nature connection, suggesting that the more aware a person is of the connection between inner bodily sensations and emotions, the more likely they can bond with nature. Given that interoceptive awareness indicates a coherent relationship with the self, including effective communication between body, mind and feelings, this process is therefore implicated in the capacity for humans to bond with nature. In sum, this present research points to the efficacy of an embodied, secure attachment with nature to help close both the human-nature disconnection chasm, and the environmental value-action gap. Theoretical and methodological implications for research and policy are discussed.

## 1 Introduction

Human-caused climate change, advanced through rapid industrialization over the past two centuries, has led to rampant biodiversity loss, extensive-reduction of wetlands and woodlands, and negative transformation of ice-free land and oceans with economic (Burke et al., 2015), social (Goldstein, 2016) and health (Smith and Myers, 2018) consequences. The global rate of nature's decline is unprecedented in human history and is directly linked to human actions, with one million species threatened with extinction and 69% of animal populations already having been lost since 1970 (Brondizio et al., 2019). This evidence suggests human complicity in the rampant loss and damage of nature, which also lays bare an ongoing coloniality, co-constitutive of processes of capitalism and imperialism (Sultana, 2022). Disconnection from nature is one of the fundamental root causes from which environmental change results (Redvers et al., 2023). The human-nature relationship, or the connectedness between humans and nature, a psychological term that captures various aspects of the human relationship to nature, has been severely damaged (Ives et al., 2017; Richardson, 2023). If the apparent dissolution of the relationship between humans and nature continues, societies will increasingly operate outside safe planetary boundaries, a concept introduced in 2009 to define the environmental limits in which humanity can safely operate (Steffen et al., 2015). Yet, the human-nature relationship is essential to mitigate climate collapse, illustrated by a recent meta-analysis of 147 correlational studies indicating that the strength of the human-nature relationship is critical for predicting resource management and sustainability (Barragan-Jason et al., 2022). The same meta-analysis also analyzed 59 experimental studies and found that environmental education leads to no effect on pro-nature behaviors, whereas people with higher nature connectedness have significantly more pro-nature behaviors, and are also significantly healthier. This evidence points to an interdigitated relationship between nature connection, pro-environmental behaviors and human health. Nature connection is linked to mindsets that value sustainability and behaviors that enhance it. Therefore, concerted effort is necessary to explicate both the root causes of this disconnection and discover leverage points for reconnection.

### 1.1 Nature connection

Empirical studies suggest nature connection can have immense benefits for humans, including improving individual wellbeing (Lambert et al., 2020), with mood (MacKerron and Mourato, 2013), cognitive (Berman et al., 2008), health (Frumkin, 2001), and longevity (Mitchell and Popham, 2008) benefits, indicative of nature's importance for overall optimal health (Aberson et al., 2000).

Recent epidemiological studies demonstrate the association between people's perceived health and availability of green space (de Vries et al., 2003; Maas et al., 2008), suggesting that the urban-rural health gap is mediated by a discrepancy in nature availability (Richardson et al., 2020). Several public health experimental studies have also discovered positive physiological benefits as a result of exposure to nature in diverse ways, including forest bathing, mindful exposure and even virtual contact (Song et al., 2016; Frost et al., 2022; Kotera et al., 2022).

Nature connection can also restore emotional and cognitive resources. In a meta-analysis of empirical research surveying 32 studies with a total of 2,356 participants, exposure to natural environments was associated with a moderate rise in positive affect and small, but consistent, decreases in negative affect (McMahan and Estes, 2015). The stress-reduction theory [SRT; (Ulrich et al., 1991)], and the satisfaction of the need to be connected to the natural world as theorized by the Biophilia hypothesis (Wilson and Kellert, 1993), could explain these results. While these findings are suggestive of nature's impact on emotional resourcing, the mechanism of emotional regulation is unclear.

Emotional regulation mediates the relationship between nature connection and happiness (Richardson and McEwan, 2018), and between nature connectedness and stress (Bakir-Demir et al., 2021), however more research is needed to understand the relationship between emotional regulation and nature connection. If nature connection is indeed primarily an emotional bond as Richardson and McEwan (2018) argues, then investigating personal factors that determine these emotional regulation capacities is potentially important.

The theoretical background of nature connection, or nature relatedness, draws on Wilson's Biophilia hypothesis, which theorizes that humans are born with an innate tendency to affiliate with nature (Wilson, 1984). Nature connection is different than exposure to greenspaces, defined as open, undeveloped land with natural vegetation (Twohig-Bennett and Jones, 2018), as well as outdoor learning environments (Harris, 2021). While simply being *amongst nature* suggests a form of passive engagement and potentially perpetuates the very objectification of nature that nature immersion attempts to correct, *nature connection* is generally concerned with the aesthetic, intellectual, cognitive, spiritual and emotional dimensions of connection, as put forward by Wilson (1984). However, the nature connection literature contains multiple and conflicting definitions of nature connection, which is also reflected in measurement.

In some cases, nature connection is defined cognitively *as being part of nature* or *self in nature* (Richardson et al., 2021; Barragan-Jason et al., 2022), while other scholars argue it is *a capacity to feel a pleasant and secure connection to nature* (Baxter and Pelletier, 2019). Still others suggest it is *an emotional and psychological connection to nature* (Richardson et al., 2021), and yet others as a function of *love and deep caring for nature* (Perkins, 2010). Nisbet et al. (2019) stress that nature relatedness is a multi-dimensional construct which captures several facets of human-nature relationships including cognition, affect and experience. Overall, nature connection scholars converge on agreeing that nature relatedness is distinct from *a general sense of connection* or environmental attitudes (Nisbet and Zelenski, 2013). Yet what exactly is nature connection?

Recent meta-analyses of the empirical nature connection studies (Tam, 2013; Whitburn et al., 2020) suggest that it is both inconclusive which measures properly capture the nature connection construct, and how divergent or convergent they are. The result is diffuse approaches to increasing so-called nature connectedness without consistency in what it is that is being targeted. Furthermore, while these definitions seem to capture the *subjective sense* of connectedness with nature and claim to operationalise a relationship between humans and nature, *the mechanism of relationship* between humans and nature remains unclear.

Empirical nature connection studies mostly test the efficacy of various intervention designs, including comparing dosage length and frequency (e.g. brief vs. extended), as well as different types of nature exposure (e.g. nature walks, noticing nature, virtual, various primes including active or passive attention, etc.), which all focus on *how to increase nature connection*. What remains missing is further explication on what this relationship comprises of.

In a study that sought to go beyond general nature contact knowledge and knowledge-based activities, specific routes to nature connectedness were examined, systematically structured around the nine values of the Biophilia hypothesis (Lumber et al., 2017). Contact, emotion, meaning, and compassion were identified as ‘routes' or pathways to increasing nature connection. Another study using data from a large national survey in the United Kingdom, revealed that *noticing nature* vs. spending time in nature, explained levels of nature connectedness (Richardson et al., 2022). In both of these examples, the *ways* to increase nature connection are more illuminated, and yet the mechanism of *relationship* between humans and nature remains muddled. If empirical research continues to only test *how* connected to nature people are but not *what* this relationship equates, important mechanisms of action as well as critical personal factors in predicting nature connectedness will go overlooked.

In addition, empirical studies suggest nature connection is not just important for wellbeing (Dean et al., 2018), but can even motivate pro-environmental behavior (Whitburn et al., 2020). In a meta-analysis of 37 independent samples from 26 studies of 13,237 individuals, a random-effects model demonstrated a positive and significant association between connection to nature and pro-environmental behaviors (Whitburn et al., 2020). This is the strongest empirical evidence in the nature connection science literature to date of the link between nature connection and pro-environmental behaviors. With public health demands increasing for research that can meaningfully address what is called the environmental value-action gap, or the gulf between one's understanding and care of nature and a willingness to act on behalf of nature (Barr, 2006), as well as to create new ways to work together for a just and sustainable future (Redvers et al., 2023), this evidence is promising.

### 1.2 Relational attachment to nature

Therefore, to examine a *relationship* with nature, then understanding both the human motivation for bonding with nature through the lens of relational attachment theory could be efficacious, in addition to isolating the core emotional regulation capacity. Secure relational attachment as first theorized by Bowlby (1969), provides a basis for healthy self-relating and the capacity for intimate relationships throughout the lifespan (Bowlby, 1969; Ainsworth, 1978; Mikulincer and Shaver, 2003). Schaller (2007) believed that secure attachment is important for such a diverse range of meaningful outcomes that, a “sense of secure attachment may be the psychological equivalent of a broadband antibiotic” (p. 191). Research by Mikulincer and Shaver (2003) suggests that when attachment security is made salient, it creates not just a sense of interpersonal attachment, but security in general.

Building on the half-century of research since Bowlby's attachment theory was put forward, attachment security in relation to nature connectedness is worth investigating as an avenue to get closer to the processual *relationship* between humans and nature. In an empirical study to investigate the psychological determinants of place attachment, which is defined as “the bonds that humans share with particular settings” (Nisa et al., 2020), attachment security was induced, which was found to increase the strength of place attachment, particularly in individuals with insecure attachment styles (Nisa et al., 2020). In the first study to examine if a sense of connectedness deriving from secure attachment could indeed extend to external environments, Nisa et al found that the ability for humans to connect with humans is crucial to understanding how humans bond with place. Over four studies, results indicate that attachment style is associated with the strength of place attachment (Nisa et al., 2020). While Nisa et al.'s research suggests a link between attachment security and attachment to place, more research is needed on attachment security and attachment to nature, specifically on the role of secure attachment to mediate the relationship between potential personal factors and nature connectedness.

To further this point, a review on personal and social influences on environmental concern indicated that personal identification with a place is a critical predictor of environmental concern and behavior (Gifford and Nilsson, 2014), therefore suggesting that the *role of bonding* takes a part in explaining individuals' responses to environmental problems. Therefore, it is feasible to investigate a motivational framework toward connecting with nature that is partially satisfying of the interpersonal need to attach, drawing upon the belongingness hypothesis (Baumeister and Leary, 1995), and expressed via individual differences in attachment style.

Since the bonds humans develop with other humans are consequential to how humans bond with place, this present research seeks to understand if human attachment is likewise related to the processual mechanism of bonding with nature.

The literature on nature connection supports the importance of interpersonal processes within the human-nature bond, exploring in multitudinous ways that a close relationship with nature is a basic human psychological need from cognitive, emotional and physiological dimensions, and one that must be filled in order to experience increased wellbeing (Baxter and Pelletier, 2019; Richardson et al., 2021). Baxter and Pelletier (2019) argue that this psychological need is only satisfied by *in vivo* immersive experiences in nature that are pleasant.

However, the nature connection literature often defines nature connectedness as *nature immersion* and *nature exposure* (Baxter and Pelletier, 2019), ignoring interpersonal relational process mechanisms. More specificity is needed to understand the interpersonal processes within the human-nature bond.

One phenomenological study explored participants' experiences with the natural world and found that the natural world was indeed experienced as a primary attachment figure, a secure base, and as embodied (Schweitzer et al., 2018). These results support the need for more research into relational attachment and embodied factors of nature relatedness. In sum, explicating the human-nature relationship *as a function of a secure attachment to nature*, and examining it as a personal factor of nature connectedness, would fill the gap in the nature connection science literature related to the mechanism of human-nature bonding.

### 1.3 Interoceptive awareness as emotional regulation mechanism

Considering that emotional regulation is important in maintaining secure human relational attachment (Ferraro and Taylor, 2021), emotional regulation could be likewise critical in the human-nature attachment. Health science and biomedical literature offers a framework to understand the role of the body in emotional regulation, through the construct of *interoceptive awareness* (body awareness). Interoceptive awareness refers to the processing and central representation of afferent internal bodily signals (Critchley and Garfinkel, 2017). Interoceptive awareness is based on an *interoceptive predictive processing framework* in which emotional feeling states arise from physiological changes in the body. Increased body awareness improves accurate detection of emotional states and boosts regulation of them (Critchley and Garfinkel, 2017; Quadt et al., 2018).

Interoceptive awareness is derived from peripheral theories of emotion (James, 1884), and has been found to have special efficacy to reducing anxiety (Dunn et al., 2010) through improving emotional regulation (Füstös et al., 2013; Dunne et al., 2021), is integral to higher-order cognition (Khalsa et al., 2017), and is thought to facilitate regulation of an integrated sense of self by decreasing distress will be (Price and Hooven, 2018). According to Price and Hooven (2018), when applying reappraisal as an emotion regulation strategy, interoceptive awareness facilitates the downregulation of affect-related arousal.

These findings suggest that the more aware a person is of ongoing bodily processes, the more successful this person's emotion regulation in response to negative affect. A correlational study found that interoceptive awareness and dispositional mindfulness, which is thought to encourage insight into the relationship between mind and body, are tightly interwoven and partly overlapping constructs, and that both are independently linked to psychological wellbeing (Hanley et al., 2017). Yet while interoception has also been seen to improve emotional regulation amongst autistic populations (Nord and Garfinkel, 2022), and a putative target for novel interventions to address neural activation in specific psychiatric disorders (Nord et al., 2021), there has been no published research into the potential interconnection between body awareness and nature connection.

Even though nature connection studies have investigated the relationship between physiological health and nature connection, body-based awareness has been measured only as physiological health indicators. For example, the benefits of “forest bathing” (therapeutic restorative-effects of physiological relaxation in a forest or natural environment) have been found to improve physical and mental health, defined as a decrease in the most prevalent mental health disorders (e.g., depression, anxiety and stress) (Song et al., 2016; Kotera et al., 2022). Forest bathing is a type of nature connection practice dependent on immersing oneself in nature using the senses (Song et al., 2016; Timko Olson et al., 2020), which could be argued increases somatosensory areas and interoceptive pathways (Medeiros et al., 2023). However, the engagement of one's senses in these interventions is via an external focus vis à vis paying attention to internal bodily sensations. In a recent meta-analysis of the human-nature relationship as a pathway to sustainability, 59 experimental studies were analyzed that attempted to increase nature connectedness (Barragan-Jason et al., 2022). Out of the six types of experimental designs identified, none primed participants to pay attention to *inner bodily sensations*.

Given that body awareness is connected to emotional regulation (Khalsa et al., 2017), and emotional regulation has been cited as important for nature connection (Korpela et al., 2018; Richardson and McEwan, 2018), the role of body awareness in the human-nature relationship deserves attention. Richardson and McEwan (2018) linked *emotional regulation* to the wellbeing benefits of nature connectedness, highlighting the role of affect regulation. Yet mechanisms that predict emotional regulation are not captured in existing nature connection studies. Common predictors in nature connection studies include demographic information, nature-exposure condition, and individual differences like mind-attribution, the big five personality traits (Tam, 2015), spiritual and religious attitudes (Preston and Shin, 2022), personal intentions (Sparks et al., 2014), and eudaimonic values (Shin et al., 2022). Empirical studies in nature connection therefore investigate what *can increase nature connectedness*, but not necessarily *what personal factors are essential* for nature connectedness to occur.

### 1.4 Preliminary study development

The theoretical framework of this present study was informed by the results of an interpretative phenomenological analysis (IPA) study. The methodology of analysis was followed step-by-step as outlined by Smith et al. (2022). In-depth interviews lasting 1.5 h were conducted with six environmental activists to better understand the phenomenology of their human-nature bond. Considering these people were already utilizing their professional lives to address climate collapse, understanding their relationships with nature could be efficacious to illuminating the experience of the human-nature bond, revealing gaps and areas for further research. In addition, the majority of nature connection research is conducted amongst mainly Western, Educated, Industrialized, Rich and Democratic nations [WEIRD; (Henrich et al., 2010)]. I sought to correct for this in part by orienting this research first in the perspectives of Black, Indigenous and People of Color in an interpretative phenomenological analysis study.

The ramifications of the separation between humans and nature have wrought cumulative harms to biodiversity, lands and waters, intertangled with the interlocking oppressions of race, class, gender and other axes (Sultana, 2022). The IPA study asked: what is an equitable relationship between humans and nature that can challenge the normalization of climate breakdown and meaningfully address it?

Four group experience themes emerged:

- Body awareness: the experience of the body is a vehicle of sensory connection and communication with nature.
- Relational attachment: the emotional and intimate close bond with nature occurs through the attachment mechanism.
- Entangled identity and sacred cosmology: widening individual identities beyond the locus of the individual “self,” into entanglement is precipitated on anthropomorphic and animistic tendencies.
- Intersectional environmentalism: a close bond with nature motivates intersectional justice as an experiential act, rooted in reciprocal relationship.

Since interpretative phenomenological analysis studies are based on a discrete number of cases and not meant for generalizability (Smith et al., 2022), it was necessary to examine these dimensions with other methods suited for understanding individual differences in line with a broader research aim. The following study can be seen as inspired by these IPA results. Two dimensions, body awareness and relational attachment, were taken forward in the following study. The entangled identity theme was not taken forward since there is already ample evidence in the existing literature to the importance of an interdependent self-construal in a bond with nature. For example, the widely-used Inclusion of Nature in the Self Scale (INS) developed by Schultz (2002). A meta-analysis found that INS had a small but significant effect size (*r* = 0.0.25) in predicting pro-environmental behavior (Whitburn et al., 2020). The fourth theme, intersectional environmentalism, requires developing new scale items to reflect these behaviors, which are not currently captured in pro-environmental behavior or sustainability behavior scales. This author has done so and that scale is in development.

### 1.5 This present research

The statement of purpose for this research is as follows: are body awareness and relational attachment critical personal factors that explicate the human-nature relationship? Furthermore, does a relationship with nature benefit not only human health but influence pro-environmental behaviors and human wellbeing?

This present research seeks to utilize structural equation modeling to investigate support for the following redefinition of nature connectedness: *an embodied, secure relationship with nature with positive personal and collective wellbeing consequences*.

An online survey research design was chosen as a first step to validate the above definition of nature connectedness given its robustness in estimating inter-relationships between constructs.

One way to bridge the disconnection between humans and nature is through *restoring* an equal-status relationship between humans and nature. The science of nature connectedness gets close to this aim, but this present research argues, does not yet go far enough.

This research agenda is split into the following hypotheses:

- Interoceptive awareness predicts nature connectedness.
- Interoceptive awareness predicts nature connection even after controlling for covariates.
- A revised theoretical model of nature connection indicates support for a reciprocal, embodied, secure relationship with nature.
- Relational attachment mediates the relationship between interoceptive awareness and nature connection.

**Figure 1:**
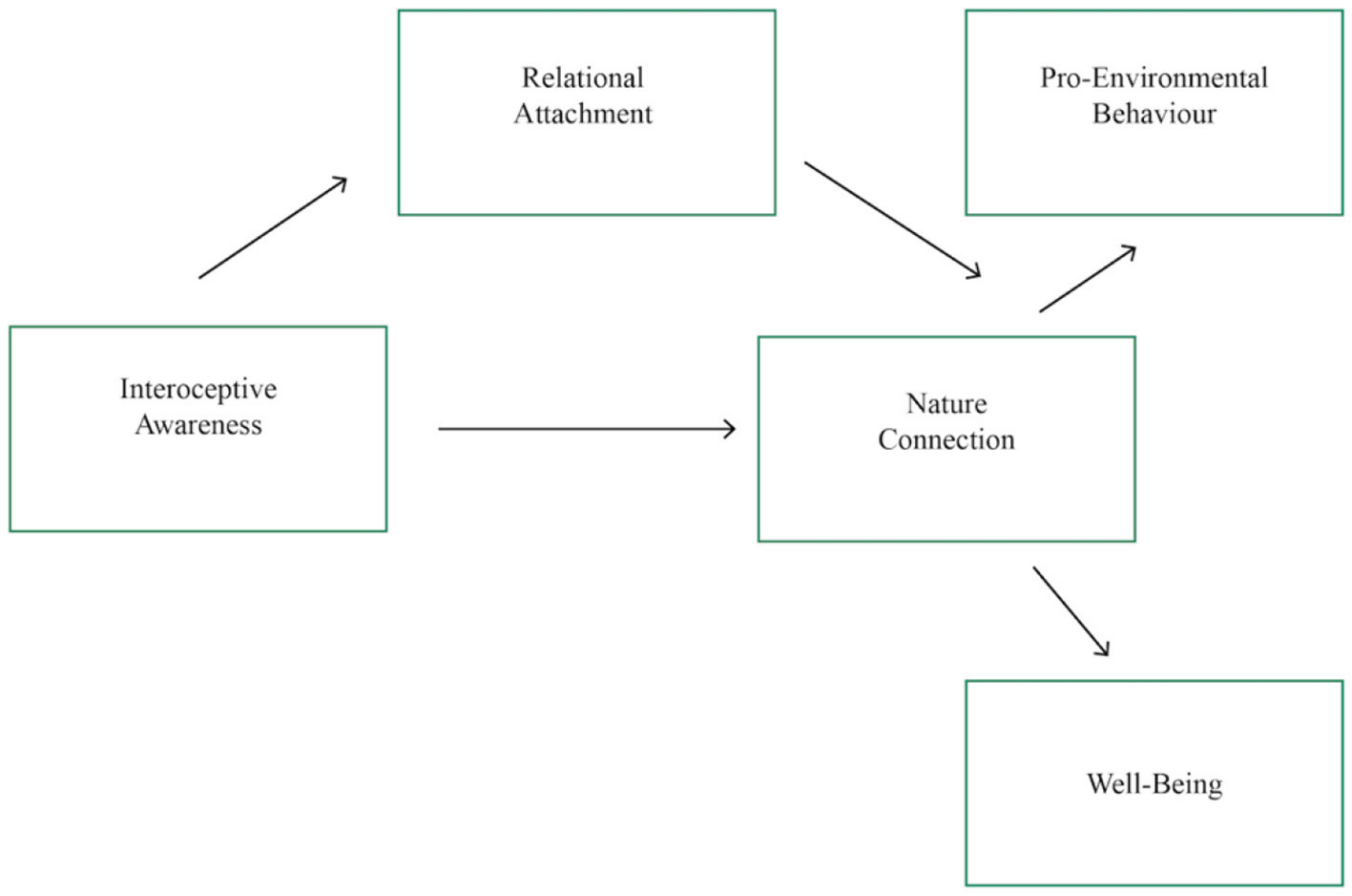
Hypothesized relationships between constructs. This figure theorizes how the personal factors of Interoceptive Awareness and Relational Attachment predict Nature Connection and subsequent wellbeing and pro-environmental behavior which build on previous literature (Martin et al., 2020).

The proposed structural model under investigation in this study is below (see Figure 1). Based on previous studies, a relationship is expected between nature connection and pro-environmental behavior (Mackay and Schmitt, 2019), as well as from nature connection to wellbeing (Martin et al., 2020). Interoceptive awareness is expected to predict nature connection (Hypothesis 1), even when controlling for confounds (Hypothesis 2) but that not all dimensions of interoceptive awareness will be significant. The revised structural model of nature connection (Hypothesis 3) will enhance theoretical precision of the human-nature bond and its impacts and support this study's definition of nature connectedness. Relational attachment will mediate the relationship between interoceptive awareness and nature connection (Hypothesis 4). This data is correlational and so causality claims should be considered with caution.

## 2 Materials and methods

### 2.1 Participants and procedure

Participants included a convenience sample from the United Kingdom and the United States recruited on Prolific, an online research platform, who passed two attention checks and gave informed signed consent (*N* = 299). The final sample (61.20% female) ranged from 19 to 93 years old (*M* = 39.75*, SD* = 15.41) with varying levels of education; high school (15.41%), undergraduate (75.90%) and graduate (8.70%). Participants were mostly White (73.6%), and some rated religion as extremely important (28.1%).

For those who had childhood contact with nature, 25.75% rated it as extremely important (*M* = 3.6*, SD* = 1.12).

For full demographic summary statistics see Table 1.

**Table 1:** Demographic summary statistics of sample.

**Summary statistics**	**Summary**
N	299
**Marital status**	
Single, never married	147 (49.2%)
Married or domestic partnership	104 (34.8%)
Widowed, divorced or separated	48 (16.1%)
**Ethnicity**	
White	220 (73.6%)
Hispanic	31 (10.4%)
Black/mixed	20 (6.7%)
Other	28 (9.4%)
**Religion**	
Christian	88 (29.4%)
Catholic	22 (7.4%)
Eastern Spirituality	14 (4.7%)
Jewish	7 (2.3%)
Atheist	34 (11.4%)
Agnostic	50 (16.7%)
Other	84 (28.1%)
**Religion importance**	
Extremely important	84 (28.1%)
Very important	77 (25.8%)
Moderately important	56 (18.7%)
Not really important	42 (14.0%)
Not at all important	40 (13.4%)

Percentage frequency in parenthesis out of sample.

### 2.2 Measures

All outcome measures, including means and standard deviations, are listed in Table 2. Independent variable measures are listed in Table 3 including subdimensions. Confound variables including means and standard deviations are in Table 4. The measures are listed below including Cronbach alpha reliability statistics.

**Table 2:** Overview of all items used as outcome variables, with descriptive statistics.

**Outcome variables**	**M *(SD)***
N	299
**Love and care for nature**	
I feel joy just being in nature	5.589 (1.354)
I feel that closeness to nature is important for my wellbeing	5.358 (1.536)
When I am close to nature, I feel a real sense of oneness with nature	5.268 (1.502)
I feel content and somehow at home when I am in unspoilt nature	5.375 (1.452)
I feel a deep love for nature	5.575 (1.382)
I often feel emotionally close to nature	5.060 (1.613)
When I spend time in unspoilt nature I feel that my day- to-day worries seem to dwindle away in the face of the wonder of nature	5.284 (1.450)
Protecting the wellbeing of nature for its own sake is important to me	5.686 (1.259)
I feel spiritually bound to the rest of nature	4.803 (1.764)
I feel a personal sense of interconnectedness with the rest of nature	5.127 (1.598)
I often feel a sense of awe and wonder when I am in unspoilt nature	5.645 (1.359)
I often feel a strong sense of care toward the natural environment	5.452 (1.339)
I need to have as much of the natural environment around me as possible	4.903 (1.585)
When in natural settings I feel emotionally close to nature	5.264 (1.565)
I enjoy learning about nature	5.880 (1.223)
**Pro-environmental behaviors**	
I signed a petition about an environmental issue.	2.823 (1.898)
I intentionally voted for a candidate in an election because of their environmental platform.	3.358 (2.016)
I encourage other people to protect the environment.	3.960 (1.745)
I compost my kitchen waste.	2.575 (1.929)
I usually buy eco-friendly products and brands	3.829 (1.422)
I choose to walk or cycle instead of using my car when I can	3.395 (1.954)
I volunteer to help care for the environment	2.371 (1.605)
**Vitality**	
I feel alive and vital.	4.405 (1.677)
Sometimes I feel so alive I just want to burst.	3.318 (1.660)
I have energy and spirit.	4.478 (1.672)
I look forward to each new day.	4.395 (1.722)
I nearly always feel alert and awake.	5.431 (6.277)
I feel energized.	3.873 (1.621)

SD, standard deviation.

**Table 3:** Overview of all items used for interoceptive awareness including subdimensions with descriptive statistics.

**Interoceptive awareness**	**M *(SD)***
*N*	299
**Noticing**	
When I am tense I notice where the tension is located in my body.	3.231 (1.076)
I notice when I am uncomfortable in my body.	3.706 (1.068)
I notice where in my body I am comfortable.	3.268 (1.142)
I notice changes in my breathing, such as whether it slows down or speeds up.	3.355 (1.145)
**Not-distracting**	
I ignore physical tension or discomfort until they become severe.	3.274 (1.086)
I distract myself from sensations of discomfort.	3.201 (1.033)
When I feel pain or discomfort I try to power through it.	2.579 (1.008)
I try to ignore pain.	2.910 (1.097)
I push feelings of discomfort away by focusing on something else.	2.900 (0.974)
When I feel unpleasant body sensations, I occupy myself with something else so I don't have to feel them.	3.077 (0.992)
**Not-worrying**	
When I feel physical pain I become upset.	3.331 (1.127)
I start to worry that something is wrong if I feel any discomfort.	3.298 (1.112)
I can notice an unpleasant body sensation without worrying about it.	2.622 (1.046)
I can stay calm and not worry when I have feelings of discomfort or pain.	2.819 (1.043)
When I am in discomfort or pain I can't get it out of my mind.	3.468 (0.980)
**Attention-regulation**	
I can pay attention to my breath without being distracted by things happening around me.	2.843 (1.089)
I can maintain awareness of my inner bodily sensations even when there is a lot going on around me.	2.896 (1.058)
When I am in conversation with someone, I can pay attention to my posture.	2.779 (1.083)
I can return awareness to my body if I am distracted.	3.017 (1.008)
I can refocus my attention from thinking to sensing my body.	2.950 (1.024)
I can maintain awareness of my whole body even when a part of me is in pain or discomfort.	2.839 (1.040)
I am able to consciously focus on my body as a whole.	3.043 (1.133)
**Emotional awareness**	
I notice how my body changes when I am angry.	3.117 (1.183)
When something is wrong in my life I can feel it in my body.	3.114 (1.199)
I notice that my body feels different after a peaceful experience.	3.508 (1.202)
I notice that my breathing becomes free and easy when I feel comfortable.	3.495 (1.222)
I notice how my body changes when I feel happy/joyful.	3.465 (1.193)
**Self-regulation**	
When I feel overwhelmed I can find a calm place inside.	2.522 (1.056)
When I bring awareness to my body I feel a sense of calm.	2.759 (1.109)
I can use my breath to reduce tension.	2.843 (1.071)
When I am caught up in thoughts, I can calm my mind by focusing on my body/breathing.	2.709 (1.120)
**Body listening**	
I listen for information from my body about my emotional state.	2.776 (1.173)
When I am upset, I take time to explore how my body feels.	2.401 (1.090)
I listen to my body to inform me about what to do.	2.632 (1.114)
**Body trust**	
I am at home in my body.	3.281 (1.202)
I feel my body is a safe place.	3.298 (1.216)
I trust my body's sensations.	3.411 (1.097)

SD, standard deviation.

**Table 4:** Overview of all items used as confound variables with descriptive statistics.

	**Summary**
*N*	299
**Meaningful psychedelic experience**	
Extremely important	32 (10.7%)
Very important	27 (9.0%)
Moderately important	27 (9.0%)
Not really important	23 (7.7%)
Not at all important	17 (5.7%)
Never had psychedelic experience	173 (57.9%)
**Meaningful mystical experience**	
Extremely important	2 (0.7%)
Very important	18 (6.0%)
Moderately important	35 (11.7%)
Not really important	34 (11.4%)
Not at all important	37 (12.4%)
Never had mystical experience	173 (57.9%)
**Frequency spending time in nature as a child**	
Daily	97 (32.4%)
Multiple times a week	118 (39.5%)
Once a week	34 (11.4%)
Once a month	11 (3.7%)
Few times a year	24 (8.0%)
Rarely	15 (5.0%)
**Openness**	
I have a rich vocabulary.	3.886 (1.105)
I have difficulty understanding abstract ideas.	2.201 (1.184)
I have a vivid imagination	4.114 (1.030)
I am not interested in abstract ideas.	2.187 (1.217)
I have excellent ideas.	3.873 (0.933)
I do not have a good imagination.	2.067 (1.299)
I am quick to understand things.	3.870 (0.959)
I use difficult words.	3.237 (1.176)
I spend time reflecting on things.	4.341 (0.881)
I am full of ideas.	4.013 (1.003)

Percentage frequency in parenthesis out of sample.

#### 2.2.1 Interoceptive awareness

Interoceptive sensibility is defined as the self-perceived tendency to focus on interoceptive stimuli (Desmedt et al., 2022). According to Khalsa et al. (2017), this construct is well-captured by the 37-item scale, Multidimensional Assessment of Interoceptive Awareness (MAIA) (Mehling et al., 2012, 2018). The MAIA includes the following eight subdimensions; (1) Noticing (e.g. “I notice where in my body I am comfortable”), (2) Not-distracting (e.g. “I distract myself from sensations of discomfort”), (3) Not-worrying (e.g. “When I feel physical pain I become upset”), (4) Attention regulation (e.g. “I can return awareness to my body if I become distracted”), (5) Emotional awareness (e.g. “I notice how my body changes when I am angry”), (6) Self-regulation (e.g. “I can use my breath to reduce tension”), (7) Body listening, (e.g. “I listen to my body to inform me about what to do”), (8) Trusting (e.g. “I am at home in my body”). Responses included nine reverse-key items and were rated on a scale from 1—*Never* to 5—*Always* (α = 0.91).

#### 2.2.2 Nature connection

The 15-item Love and Care for Nature Scale (LCN) was chosen due to the explicitly emotional dimensions of love and deep caring it captures which is in line with the theoretical proposition of this study (e.g. “I feel a deep love for nature; Perkins, 2010). The LCN scale has been used consistently throughout the nature connection literature (Tam, 2013). Responses included no reverse-key items and were rated on a scale from 1—*Strongly disagree* to 7—*Strongly agree* (α = 0.97).

#### 2.2.3 Wellbeing

Eudaimonic wellbeing is the most consistently enhanced form of wellbeing according to the nature connection literature (Pritchard et al., 2020). Among the various dimensions of eudaimonic wellbeing (such as positive affect and life satisfaction), vitality is most strongly associated with nature connectedness (Capaldi et al., 2014). Therefore, the 6-item Vitality Scale was used to measure eudaimonic wellbeing (“I feel alive and vital”; Ryan and Frederick, 1997). Responses included no reverse-key items and were rated on a scale from 1—*Not at all true* to 7—*Very true* (α = 0.90).

#### 2.2.4 Pro-environmental behavior

An 8-item scale was used to assess both personal and public sphere PEBs, according to a coherent theory on pro-environmental behavior (Stern, 2000), with questions from Martin et al. (2020). Personal sphere pro-environmental behaviors included items related to recycling, conserving gasoline, buying ethical fashion, plastic use and more. Public sphere PEBs included items related to lobbying, voting, veganism and more. The scale was kept as one dimension instead of two, due to low reliability if kept separate. Responses included no reverse-key items and were rated on a scale from 1—*Never* to 7—*Always* (α = 0.81).

#### 2.2.5 Relational attachment humans

The proposed mediator is both human-human and human-nature relational attachment. The 9-item Experiences in Close Relationships measure was utilized (Wei et al., 2007), which is considered to have the greatest precision and validity in measuring relational attachment (Fraley et al., 2000). The scale investigates approach-avoidance (e.g. “I don”t feel comfortable opening up to others”), approach-anxiety (e.g. “I”m afraid that other people may abandon me”), and security in human-human relationships (e.g. “It helps to turn to people in times of need”). The first four items are reverse-keyed and were rated on a scale from 1—*Strong disagree* to 7—*Strongly agree* (α = 0.86).

#### 2.2.6 Relational attachment nature

In addition, per the authors admonition, I adapted the relational attachment scale to substitute words of nature in order to measure attachment to nature which became a secondary measure. The scale investigates human-nature approach-avoidance (e.g. “I don”t feel comfortable getting close to nature”), human-nature approach-anxiety (e.g. “I”m afraid that nature may abandon me”), and human-nature relational security (e.g. “I am supported by nature”). The first four items are reverse-keyed and were rated on a scale from 1—*Strong disagree* to 7—*Strongly agree* (α =0.82).

#### 2.2.7 Covariates

Lastly, to control for potential confounds, I included measures of traits and experiences suggested by previous literature; childhood contact with nature (Capaldi et al., 2014), certain psychedelic use including psilocybin (Lyons and Carhart-Harris, 2018; Forstmann et al., 2023), previous mystical experience (Paterniti et al., 2022) and trait openness to experience from the Big 5 personality test (Tam, 2013). These covariates are all predictive of nature connectedness. Accounting for the potential shared variance of these measures allows better isolation of the role of interoceptive awareness in predicting nature connection.

Openness to experience (McCrae and Sutin, 2009) was reliable (α = 0.84) and included 10-items rated on a scale from 1—*Strong disagree*, to 5—*Strongly agree*. See Table 4 for descriptive statistics of the confounds.

### 2.3 Analytic approach

Structural Equation Modeling (SEM) allows estimation between a large number of independent variables and more than one dependent variable at the same time, a superior technique compared to traditional mediation analysis, and useful for understanding inter-related structural models (Mehmetoglu and Jakobsen, 2017, p. 294). Further, due to SEM being correlative and suggestive of frameworks, not causality, it fits the overall research paradigm of this study.

## 3 Results

### 3.1 Validation and measurement quality

Before beginning the SEM analysis, Principle Component Factor Analysis (PCF), part of Factor Analysis (FA), was performed to verify the measurement quality of the constructs for the eventual SEM model (Mehmetoglu and Jakobsen, 2017). This was done as an exploratory first step which built to the eventual Confirmatory Factor Analysis (CFA). This step verifies the validity of the latent constructs before using them as independent or dependent variables in the model.

The Interoceptive Awareness (IA) latent variable was analyzed in Stata (Version 18), which indicated eight principle components. An extraction method based on principal component analysis and the promax rotation method with Kaiser normalization was executed, since the factors should be correlated. See Table 5 for generated factor scores from taking the average of the variables expressing each factor for the eight factors, as well as alpha coefficients to indicate the reliability of the summated subdimension scales (Mehmetoglu and Jakobsen, 2017, p. 287). This corroborated the literature which argues for eight subdimensions of the Interoceptive Awareness scale (Mehling et al., 2018). By running the *pcf* command on the remaining constructs, one factor was indicated for each scale, according to Kaiser criterion, which fits the theory of those constructs.

**Table 5:** Generated factor scores and alpha coefficients for each dimension of interoceptive awareness factors.

**Factor**	***M***	**SD**	**Min**	**Max**	**Cronbach alpha**
N					299
Factor 1: Noticing	3.39	0.85	1	5	0.77
Factor 2: Non-distracting	2.99	0.77	1	5	0.84
Factor 3: Not-Worrying	3.11	0.77	1	5	0.77
Factor 4: Attention-Regulation	2.91	0.83	1	5	0.89
Factor 5: Emotional Awareness	3.34	0.95	1	5	0.85
Factor 6: Self-Regulation	2.71	0.91	1	5	0.86
Factor 7: Body Listening	2.60	0.99	1	5	0.85
Factor 8: Body Trust	3.33	1.06	1	5	0.88

M, mean; SD, standard deviation.

Secondly, the suitability of the dataset was determined including the value of the determinant using the Kaiser-Meyer-Olkin (KMO) measure of sampling adequacy. KMO values between 0.8 and 1.0 indicate the sampling is adequate (Shrestha, 2021). The Interoceptive Awareness scale had a KMO of 0.89, indicating adequacy. Thirdly, appropriateness of the data set was tested for a functioning factor analysis with the Bartlett's test of sphericity. If the Bartlett's test of Sphericity is highly significant at p < 0.001, this indicates that that the correlation matrix has significant correlations among at least some of the variables (Shrestha, 2021).

The results of KMO tests for all latent constructs are in Table 6. The Bartlett's test of sphericity was statistically significant for all latent constructs, with a *p* = 0.00. Therefore, each passed the KMO and Bartlett's test of sphericity, indicating validity for use as latent constructs in SEM.

**Table 6:** KMO statistics for latent constructs.

**Factor**	**KMO**
*N*	299
Interoceptive awareness	0.92
Relational attachment	0.83
Nature attachment	0.84
Love and care for nature	0.97
Pro-environmental behaviors	0.86
Vitality	0.89

KMO, Kaiser-Meyer-Olkin test.

### 3.2 Confirmatory factor analysis

A Confirmatory Factor Analysis (CFA) was then performed for each proposed latent variable in order to investigate the hypothesized underlying structure of the data and if it fit with the theorized latent variable measurement model. A structural equation model was estimated using maximum likelihood on each potential latent variable. Model fit evaluation and satisfactory results indicated model fit for all latent variables. All factor loadings, which show strong linear combinations of underlying indicators with the latent variables, are reported in full, along with goodness of fit statistics of χ^2^, RMSEA, Comparative Fit Index (CFI), and Tucker-Lewis Index (Tucker and Lewis, 1973) in Table 7. Although no precise standards exist for what value of indices equate to good fit, typical guidelines are that TFI and CFI should exceed 0.90. RMSEA values above.10 indicate poor model fit (Mehmetoglu and Jakobsen, 2017, p. 308). All the latent variables passed these fit indices.

**Table 7:** Factor loadings and goodness of fit statistics including Chi-squared, RMSEA, Comparative Fit Index, Tucker- Lewis and convergent/divergent validity AVE and Raykov.

**Latent variables**	***Coeff*.**	**χ^2^**	**RMSEA**	**CFI**	**TLI**	**AVE**	**Raykov**	**Alpha**
**IA: noticing**		4.190	0.000	1.0	1.0	0.50	0.80	0.84
I ignore physical tension or discomfort until they become severe.	0.48^***^							
I distract myself from sensations of discomfort.	0.69^***^							
When I feel pain or discomfort I try to power through it.	0.63^***^							
I try to ignore pain.	0.76^***^							
I push feelings of discomfort away by focusing on something else.	0.73^***^							
When I feel unpleasant body sensations, I occupy myself with something else so I don't have to feel them.	0.73^***^							
**IA: emotional awareness**		4.623	0.023	0.9	1.0	0.55	0.82	0.86
I notice how my body changes when I am angry.	0.49^***^							
When something is wrong in my life I can feel it in my body.	0.55^***^							
I notice that my body feels different after a peaceful experience.	0.82^***^							
I notice that my breathing becomes free and easy when I feel comfortable.	0.87^***^							
I notice how my body changes when I feel happy/joyful.	0.86^***^							
**IA: self-regulation**		0.121	0.000	1.0	1.0	0.56	0.80	0.86
When I feel overwhelmed I can find a calm place inside.	0.74^***^							
When I bring awareness to my body I feel a sense of calm.	0.85^***^							
I can use my breath to reduce tension.	0.66^***^							
When I am caught up in thoughts, I can calm my mind by focusing on my body/breathing.	0.73^***^							
**IA: body listening**		0.000	0.000	1.0	1.0	0.67	0.85	0.85
I listen for information from my body about my emotional state.	0.78^***^							
When I am upset, I take time to explore how my body feels.	0.89^***^							
I listen to my body to inform me about what to do.	0.76^***^							
**IA: body trust**		0.000	0.000	1.0	1.0	0.73	0.90	0.88
I am at home in my body.	0.86^***^							
I feel my body is a safe place.	0.95^***^							
I trust my body's sensations.	0.74^***^							
**Nature connection: love and care for nature**		103.477	0.051	1.0	1.0	0.69	0.96	0.97
I feel joy just being in nature	0.83^***^							
I feel that closeness to nature is important for my wellbeing	0.90^***^							
When I am close to nature, I feel a real sense of oneness with nature	0.93^***^							
I feel content and somehow at home when I am in unspoilt nature	0.83^***^							
I feel a deep love for nature	0.86^***^							
I often feel emotionally close to nature	0.88^***^							
When I spend time in unspoilt nature I feel that my day- to-day worries seem to dwindle away in the face of the wonder of nature	0.83^***^							
Protecting the wellbeing of nature for its own sake is important to me	0.70^***^							
I feel spiritually bound to the rest of nature	0.85^***^							
I feel a personal sense of interconnectedness with the rest of nature	0.90^***^							
I often feel a sense of awe and wonder when I am in unspoilt nature	0.76^***^							
I often feel a strong sense of care toward the natural environment	0.80^***^							
I need to have as much of the natural environment around me as possible	0.83^***^							
When in natural settings I feel emotionally close to nature	0.90^***^							
I enjoy learning about nature	0.59^***^							
**Relational attachment humans**		1.060	0.000	1.0	1.0	0.50	0.78	0.85
It helps to turn to people in times of need.	0.57^***^							
I usually discuss my problems and concerns with others.	0.80^***^							
I talk things over with people.	0.72^***^							
I find it easy to depend on others.	0.58^***^							
I don't feel comfortable opening up to others.	0.73^***^							
I prefer not to show others how I feel deep down.	0.64^***^							
**Relational attachment nature**		1.163	0.000	1.0	1.0	0.56	0.86	0.85
It helps to turn to nature in times of need.	0.85^***^							
I usually share my problems and concerns with the nature in a way that makes sense to me.	0.65^***^							
I am supported by nature.	0.83^***^							
I find it easy to depend on nature.	0.80^***^							
I don't feel comfortable getting close to nature.	0.56^***^							
**Vitality**		8.839	0.040	0.9	0.9	0.61	0.77	0.74
I feel alive and vital.	0.90^***^							
Sometimes I feel so alive I just want to burst.	0.65^***^							
I have energy and spirit.	0.86^***^							
I look forward to each new day.	0.80^***^							
I nearly always feel alert and awake.	0.57^***^							
I feel energized.	0.86^***^							
**Pro-environmental behaviors**		20.73	0.036	0.9	0.9	0.45	0.84	0.83
I signed a petition about an environmental issue.	0.55^***^							
I intentionally voted for a candidate in an election because of their environmental platform.	0.79^***^							
I encourage other people to protect the environment.	0.73^***^							
I compost my kitchen waste.	0.78^***^							
I usually buy eco-friendly products and brands	0.35^***^							
I choose to walk or cycle instead of using my car when I can	0.64^***^							
I volunteer to help care for the environment	0.34^***^							

Significant level, ^*^p < 0.05, ^**^p < 0.01, ^***^p < 0.001.

Internal consistency was assessed by Cronbach's alpha, also reported in Table 7. Each measure passed the adequate threshold for Cronbach's alpha of > 0.7, indicating adequate to excellent internal consistency and reliability (Shrestha, 2021). According to Kline (2016), convergent validity refers to a set of indicators designed to measure a construct, which can be tested using Average Variance Extracted (AVE). A high AVE (> 0.50) indicates a high convergent validity, therefore AVE for each construct should be at least 0.50. Since a recent paper suggests removing constructs below 0.40 (Haji-Othman and Yusuff, 2022), the pro-environmental behavior variable was retained. To indicate divergent validity, Raykov's factor reliability coefficient is provided, which computes reliability coefficients for factors with and without correlated errors (Mehmetoglu and Jakobsen, 2017, p. 287). Raykov coefficients > 0.70 are considered divergent and all latent variables passed this test which are reported in Table 7.

### 3.3 Correlations

Next, Pearson correlation coefficients were calculated to assess the effect size and statistical significance of the univariate relationship between all of the variables under consideration and are reported in full in Table 8. As expected, Interoceptive Awareness is correlated with nature connection, but only on certain dimensions. Nature connection is strongly positively correlated with Emotional Awareness (*r* = 0.51), moderately positively correlated with Self-Regulation (*r* = 0.46), Body Listening (*r* = 0.39), and Body Trust (*r* = 0.30), and maintains a small positive correlation with Noticing (*r* = 0.26), Attention Regulation (*r* = 0.23), and a small negative correlation with Non-Distracting (*r* = −0.20).

**Table 8:** Correlations between all latent variables (*N* = 299).

**Variables**	**(1)**	**(2)**	**(3)**	**(4)**	**(5)**	**(6)**	**(7)**	**(8)**	**(9)**	**(10)**	**(11)**	**(12)**	**(13)**
(1) IA: noticing	1.000												
(2) IA: non-distracting	−0.025	1.000											
(3) IA: not-worrying	−0.067	0.023	1.000										
(4) IA: attention-regulation	0.486^*^	0.003	0.144^*^	1.000									
(5) IA: emotional awareness	0.582^*^	−0.118^*^	−0.028	0.427^*^	1.000								
(6) IA: self-regulation	0.345^*^	−0.073	0.206^*^	0.565^*^	0.541^*^	1.000							
(7) IA: body listening	0.512^*^	−0.031	−0.022	0.515^*^	0.594^*^	0.581^*^	1.000						
(8) IA: body trust	0.327^*^	0.072	0.330^*^	0.536^*^	0.408^*^	0.488^*^	0.450^*^	1.000					
(9) Love and care for nature	0.261^*^	−0.195^*^	0.104	0.230^*^	0.511^*^	0.455^*^	0.383^*^	0.293^*^	1.000				
(10) Pro-environmental behaviors	0.240^*^	−0.059	−0.022	0.232^*^	0.319^*^	0.350^*^	0.270^*^	0.148^*^	0.395^*^	1.000			
(11) Relational attachment	0.105	−0.077	0.095	0.127^*^	0.228^*^	0.204^*^	0.137^*^	0.241^*^	0.232^*^	0.136^*^	1.000		
(12) Relational nature attachment	0.242^*^	−0.236^*^	0.052	0.186^*^	0.425^*^	0.389^*^	0.338^*^	0.236^*^	0.810^*^	0.393^*^	0.175^*^	1.000	
(13) Vitality	0.257^*^	0.053	0.259^*^	0.372^*^	0.317^*^	0.515^*^	0.331^*^	0.573^*^	0.335^*^	0.166^*^	0.354^*^	0.290^*^	1.000

Significant level, ^*^p < 0.5, ^**^p < 0.01, ^***^p < 0.001.

Therefore, Emotional Awareness, followed by Self-Regulation, Body Listening and Body Trust, seem to be the most important dimensions of Interoceptive Awareness in connection with a bond to nature.

Vitality follows the same pattern and is highly positively correlated with Self-Regulation (*r* = 0.52), and Body Trust (*r* = 0.57), and is positively moderately correlated with Emotional Awareness (*r* = 0.32) and Attention Regulation (*r* = 0.38).

Lastly, Relational Attachment Humans is moderately positively correlated to Body Trust (*r* = 0.24), Self-Regulation (*r* = 0.20), and Emotional Awareness (*r* = 0.23). Relational attachment to nature is highly positively correlated to Emotional Awareness (*r* = 0.43), Self-Regulation (*r* = 0.39) and Body Listening (*r* = 0.34), moderately positively correlated to Body Trust (*r* = 0.24) and Noticing (*r* = 0.24), and moderately negatively correlated to Non-Distracting (*r* = −0.24).

Overall, this suggests that the dimensions of *Emotional Awareness, Self-Regulation, Body Listening, Body Trust* and *Non-Distracting* are perhaps the most important dimensions of interoceptive awareness in relationship to nature connection. Therefore, since the other dimensions are not correlated to nature connection and they did not pass the AVE threshold test, they will be excluded from the path analysis and deemed insignificant to nature connection.

Relational Attachment Humans and Relational Attachment Nature have a weak significant correlation (*r* = 0.18). This could suggest that these constructs are tapping different aspects of human bonding and that patterns of attachment in human-human bonds vs. human-nature bonds have their own unique expressions. Below are the results per research hypothesis.

**Hypothesis 1: Interoceptive awareness predicts nature connectedness**.

I ran a structural model using maximum likelihood. The following interoceptive awareness dimensions are significantly predictive of nature connection in this structural model: Non-Distracting is significantly negatively predictive (*b* = −0.33, *SE* = 0.11, *p* = 0.00, 95% [−0.55, −0.12]), meaning the more distracted one is with their bodily sensations, the less connected to nature they will be, whereas Emotional Awareness (*b* = 0.67, *SE* = 0.13, *p* = 0.00, 95% [0.45, −0.94]), and Self-Regulation (*b* = 0.36, *SE* = 0.12, *p* = 0.00, 95% [0.13, 0.56]) are positively significantly predictive of nature connection. This indicates that Emotional Awareness and Self-Regulation increase the likelihood of being nature connected. Body Listening is nearly positively significantly predictive of nature connection (*b* = 0.12, *SE* = 0.07, *p* = 0.06, 95% [0.13, 0.56]). This indicates that the various dimensions do not function similarly in their relationship to nature connection, and not all interoceptive awareness dimensions are significantly predictive.

**Hypothesis 2: Interoceptive awareness predicts nature connection even after controlling for covariates**.

A series of linear regressions indicated that certain dimensions of interoceptive awareness do significantly predict nature connection even when controlling for previous childhood nature contact, previous psychedelic use and mystical experiences. These are as follows; Emotional Awareness (*b* = 0.39, *SE* = 0.05, *p* = 0.00, 95% [0.30, 0.49]), Self-Regulation (*b*= 0.36, *SE* = 0.05, *p* = 0.00, 95% [0.27, 0.45]), Body Listening (*b* = 0.28, *SE* = 0.05, *p* = 0.00, 95% [0.19, 0.38]), and Body Trust (*b* = 0.22, *SE* = 0.05, *p* = 0.00, 95% [0.12, 0.32]). Non-distracting was significantly negatively predictive of nature connection (*b* = 0.16, *SE* = 0.05, *p* = 0.00, 95% [−0.26, −0.60]). Emotional awareness accounted for 30% of the variance in nature connection. Other dimensions ranged from 16% variance (non-distracting), 18.5% (body trust), to 28% (self-regulation), indicating the importance of these dimensions on nature connection. These subdimensions of Interoceptive Awareness follow the same pattern as in hypothesis 1.

What this indicates is that people who possess an ability to be emotionally aware, self-regulate, listen to inner bodily sensations and trust them, while being undistracted from painful and disturbing sensations, are all likely to be more connected to nature and to develop a close bond, even when controlling for the above confounds. Further, Emotional Awareness accounts for the most variance, indicating that the ability to notice, identify and locate one's emotions is highly predictive of nature connectedness. This evidence points to the importance of a coherent bodily self to motivate a relationship with nature.

**Hypothesis 3: A revised theoretical model of nature connection indicates support for a reciprocal, embodied, secure relationship with nature**.

**Figure 2:**
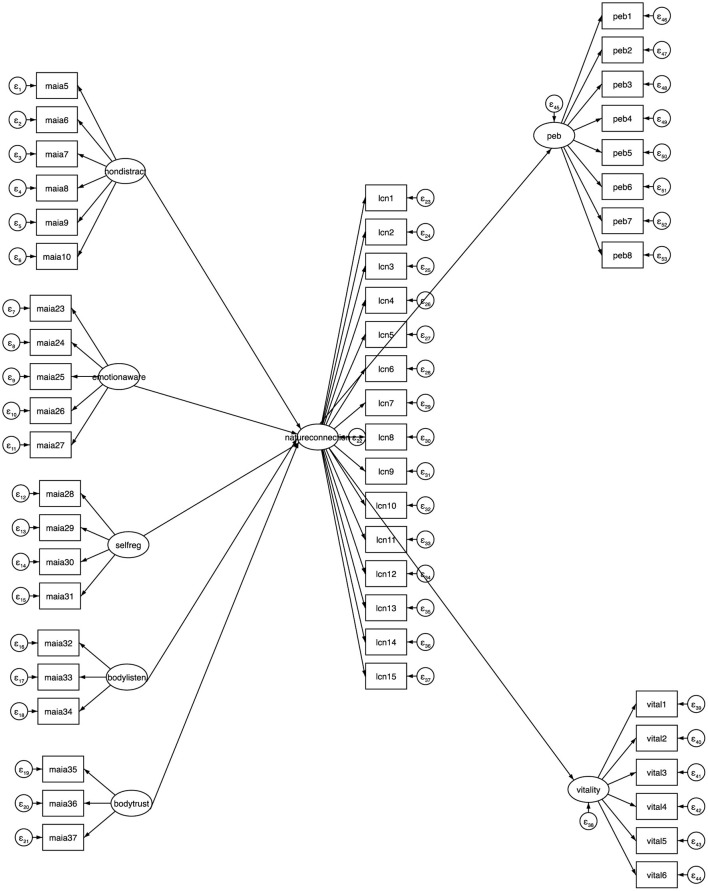
Proposed structural model of interoceptive awareness, nature connection, pro-environmental behaviors and wellbeing. Each latent variable passed validity tests. The estimated structural equation model (SEM) specifies five predictor latent variables, chosen because these Interoceptive Awareness dimensions were significant in Hypothesis 1.

A saturated, non-recursive structural model using maximum likelihood estimation and the latent variables under investigation was utilized. The model is below (see Figure 2).

**Figure 3:**
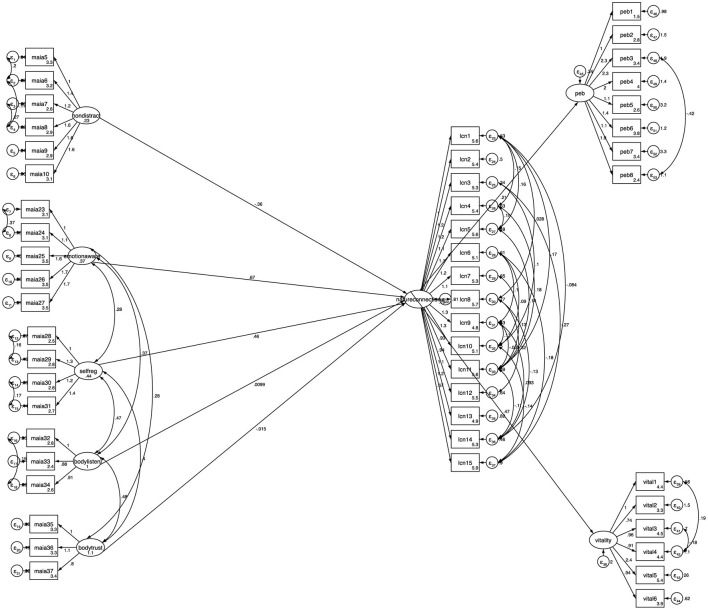
Final structural model of interoceptive awareness dimensions on nature connection, pro-environmental behaviors and wellbeing. Standardized coefficients are reported.

Table 9 reports the standardized regression weights for the model. The model identifies a significant positive relationship between the dimensions of Non-Distracting, Emotional Awareness and Self-Regulation on nature connection, which in turn significantly predicts pro-environmental behaviors and increased wellbeing. Modifications were made to the model during model fit testing and the final model used all available 299 observations and indicated good model fit (χ^2^ = 2,000.379, *p* > 0.05, CFI = 0.92, TLI = 0.91). The goodness-of-fit indices calculated for the SEM indicate the model estimated provides a good fit to the data. For the final model, see Figure 3.

**Table 9:** Structural equation model of interoceptive awareness dimensions on nature connection, pro-environmental behaviors and wellbeing.

**Latent Variable paths**	***b***	**SE**	**95% CI**
IA → Nature connection			
Emotional Awareness	0.67^***^	0.16	[0.34, 0.98]
Non-Distracting	−0.36^**^	0.13	[−0.62, −0.98]
Self-Regulation	0.46^*^	0.19	[0.07, 0.85]
Body Trust	−0.01	0.07	[−0.15, 0.13]
Body Listening	0.01	0.09	[−0.17, 0.19]
Nature Connection →			
Pro-environmental behaviors	0.21^***^	0.03	[0.14, 0.29]
Vitality	0.46^***^	0.08	[0.31, 0.62]

Standardized regression weights. Significant level, ^*^p < 0.05, ^**^p < 0.01, ^***^p < 0.001.

**Figure 4:**
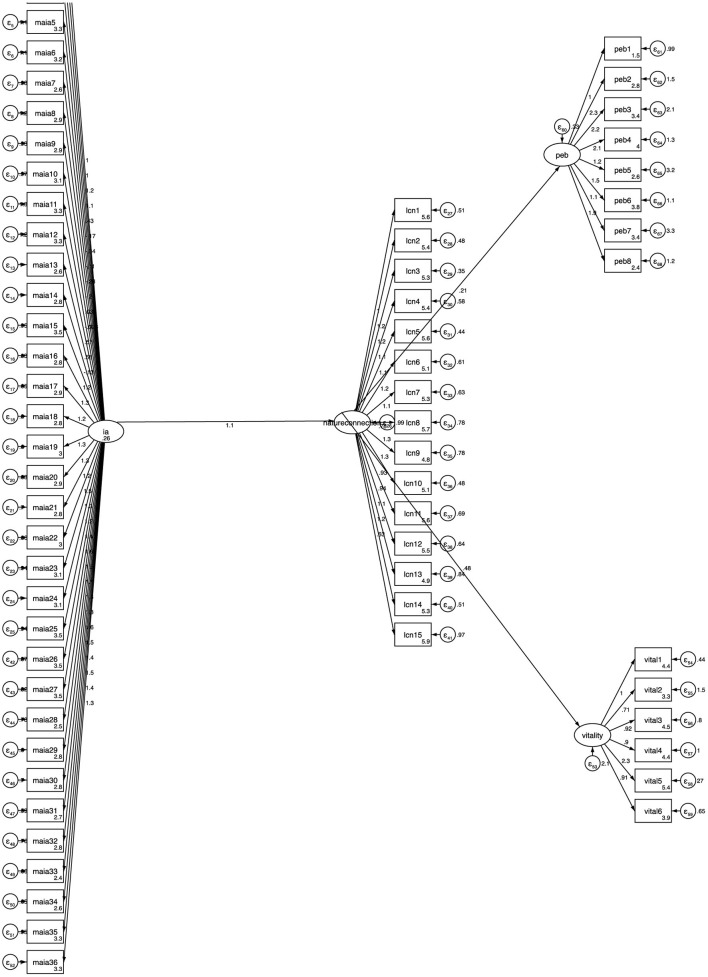
Structural model of interoceptive awareness as a single latent construct on nature connection, pro-environmental behaviors and wellbeing. Standardized coefficients are reported.

A second model was fitted that investigated the latent variable interoceptive awareness as the predictor instead of splitting this latent variable into its theoretical subdimensions. Table 10 reports the standardized regression weights for the model. Interoceptive Awareness as a latent variable is significantly predictive of nature connection, which predicts wellbeing and flourishing. Modifications were made to the model during model fit testing and the final model indicated good model fit (χ^2^ = 6,220.277, *p* > 0.05, CFI = 0.91, TLI = 0.91) (see Figure 4). Therefore, the results indicate that overall, interoceptive awareness is predictive of nature connection and subsequently increased pro-environmental behaviors and increased wellbeing, and that parsing further, the subdimensions of *emotional awareness, self-regulation and non-distracting* are responsible for this relationship.

**Table 10:** Structural equation model of interoceptive awareness, nature connection, pro-environmental behaviors and wellbeing.

**Latent Variable paths**	**Beta**	**SE**	**95% confidence interval**
IA →			
Nature connection	0.46^*^	0.19	[0.07, 0.85]
Nature Connection →			
Pro-environmental behaviors	0.21^***^	0.03	[0.14, 0.29]
Vitality	0.46^***^	0.08	[0.31, 0.62]

Beta, standardized beta regression coefficients; SE, standard error. ^*^p < 0.05. ^***^p < 0.001.

**Hypothesis 4: Relational attachment mediates the relationship between interoceptive awareness and nature connection**.

**Figure 5:**
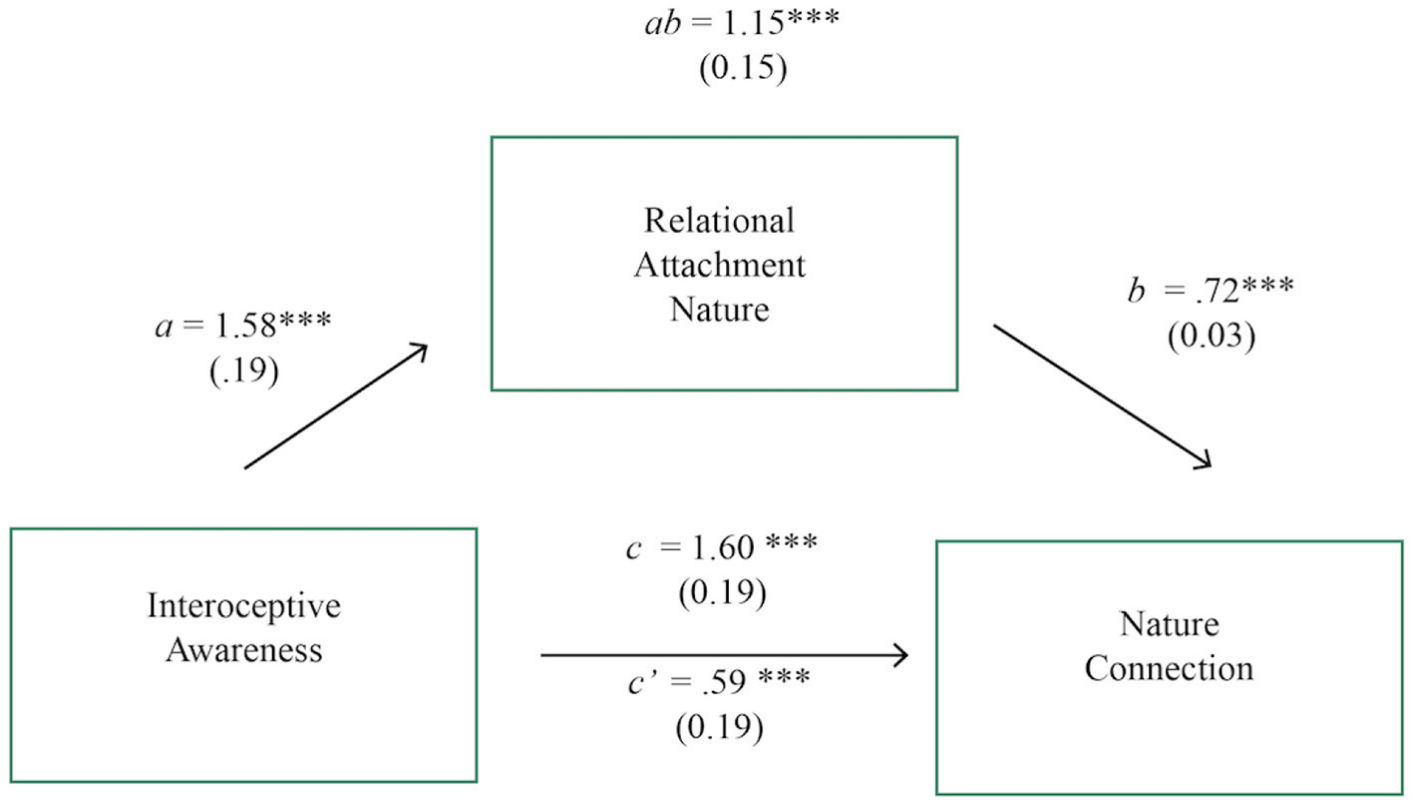
Direct, indirect and total effects of interoceptive awareness on nature connection, mediated by relational attachment to nature. Mediation diagram; a, b, c and c' are path coefficients representing unstandardized regression weights and standard errors (in parentheses). The c path coefficient represents the total effect of interoceptive awareness on nature connection. The c-prime path coefficient refers to the direct effect of the interoceptive awareness on nature connection. *ab* path is the indirect mediated effect of interoceptive awareness on nature connection via relational attachment to nature. Significant level, *p < 0.05, **p < 0.01, ***p < 0.001.

A path analysis model was estimated using maximum likelihood, testing the mediating effect of relational attachment with nature in the relationship between interoceptive awareness and nature connection. The model had good fit (χ^2^ = 442.383, *p* > 0.05, CD = 0.249) and used all available 299 observations. Results point to a statistically significant relationship between interoceptive awareness and relational attachment (see Figure 5); and respectively, between interoceptive awareness and nature connection, as well as relational attachment and nature connection. Results also indicate that the proportion of total effect mediated by relational attachment to nature was large at 76%. This indicates that the relationship between interoceptive awareness and nature connection is mostly explained by the closeness of the relational attachment with nature. The mediating effect of relational attachment with nature in the relationship between interoceptive awareness and nature connection was non-significant and so only the relational attachment to nature construct is in the mediation model.

These results indicate that secure relational attachment to nature could be more important than interoceptive awareness in predicting nature connection, or at minimum, that there is a parsimonious relationship between these constructs.

## 4 Discussion

Overall, the results lend support to the initially proposed structural theoretical model (Figure 1). Interoceptive awareness, which has not yet been investigated in the nature connection literature, indeed predicts nature connection, increased wellbeing and increased pro-environmental behaviors. Further, human-nature relational attachment plays a significant mediating role between interoceptive awareness and nature connection, indicating the importance of relational security, and lending credence to a bond with nature that is at least partially satisfying of the human need to attach and belong, occurring through the attachment-system, and built on body awareness capacities. A relationship with nature could function similarly to interpersonal relationships, built through an attachment-system that downregulates stress with the parallel benefits of positive emotions and increased wellbeing.

Finally, it is important to note that although these findings through structural and mediation models are suggestive of strong relationships, this study does not reveal causality. Instead, conceptualizing these findings as strong associations is appropriate.

The potential impact of this research includes the following; (1) to provide actionable knowledge that points to key areas that can help ameliorate the human-nature disconnection crisis; and (2) provide recommendations for nature connection interventions that could improve the growing disconnection between humans and nature and motivate environmental sustainability behaviors, thereby also addressing the environmental value-action gap.

As Botanist and Indigenous scholar Robin Wall Kimmerer writes,

Knowing that you love the earth changes you, activates you to defend and protect and celebrate. But when you feel that the earth loves you in return, that feeling transforms the relationship from a one-way street into a sacred bond (Kimmerer, 2013).

### 4.1 Body awareness is the foundation of a relationship with the earth

The cross-sectional study indicates that interoceptive awareness significantly predicts increased nature connectedness, which can be explained by its emotional regulatory capacity. *Interoceptive awareness* is the processing and central representation of afferent internal bodily signals (Critchley and Garfinkel, 2017), which leads to a coherent relationship with the self, defined as effective communication between the body, mind and emotions (Price and Hooven, 2018). People who are able to identify, assess and appraise internal bodily signals likewise experience improvement in emotional and sensory awareness, a decrease in distress, and an improvement in emotional regulation (Price and Hooven, 2018).

Of the eight dimensions of interoceptive awareness (Mehling et al., 2012), only several significantly predict nature connection; (1) non-distraction, (2) emotional awareness, and (3) self-regulation, and to a lesser degree, body trust and body listening. Perhaps these dimensions address the core capacities of emotional literacy and regulation given that they are mid-level constructs in the overall interoceptive awareness progressively-built model. Considering the nature connection literature has already well-established that the most important predictor of nature connection is an emotionally-driven bond (Richardson, 2023, p. 58), and that nature connection predicts wellbeing and pro-environmental behaviors (Whitburn et al., 2020), these findings point to a key antecedent of this bond: interoceptive awareness. Thus, the results suggest that interoceptive awareness offers something akin to a set of building blocks to nature connectedness; by not being distracted by painful sensations, possessing emotional awareness, and having the ability to regulate sensations, bonding with nature is more likely.

Emotional awareness and self-regulation were the two most highly significant dimension out of the eight, as well as holding the most variance, suggesting the dual role of both simply noticing changes within one's inner state (e.g. “I notice that my body feels different after a peaceful experience”), as well as regulating them (e.g. “I can use my breath to reduce tension”), can increase the likelihood of connecting to nature. This could be due to the dimensions' similarity to mindfulness capacities (enhancing present moment awareness).

In the effort by many researchers and interdisciplinary fields of study to understand how to reconnect humans and nature, this research therefore suggests that focusing on individual capacities for interoceptive awareness is a currently overlooked, yet critical factor. Therefore, interoceptive awareness can be understood as an entry point to a shared sensory language with more-than-human life that increases emotional regulation and subsequent bonding with nature.

To underscore the importance of interoceptive awareness as a personal factor of nature connectedness, confounds which have been previously investigated as important explainers of individual differences in nature connection, did not erase the significant relationship between interoceptive awareness on nature connection. These are as follows: previous mystical experience, psychedelic experience, childhood contact with nature and openness.

Returning to this research's philosophically phenomenological roots, embodied sensory awareness is synonymous with philosopher Merleau-Ponty (1974) conceptualization of sensation in his *Phenomenology of Perception*. Becoming reacquainted with the breathing, sensing body opens the perceptual capacity to frequent the sensorial dimension of experience in which humans are corporeally embedded. Inhabiting the body's language of sensations in an ontological manner serves to bridge the mind-body split by reconnecting humans and nature not as a matter of utility, but as *a relationship* in an ontological necessity. Such a framing binds embodiment, nature and experience together in a shared reality.

### 4.2 A relationship with nature can function as a secure relational attachment

The human-nature bond parallels human relational attachment and security-based bonding processes. Secure attachment with nature mediated the relationship between interoceptive awareness and nature connectedness, and significantly explained that relationship, indicating the importance of relational attachment with nature as theorized by Bowlby (1979). Further, the model predicted subsequent wellbeing and pro-environmental behaviors, thus indicating the importance of a secure attachment to nature in the overall wellbeing and altruism model.

These findings suggest that the relational attachment-system, which has been studied copiously in human-human relationships (Mikulincer and Shaver, 2003; Shaver et al., 2016), attenuating threat through the availability of an “attachment figure,” creating a “safe haven,” and a “secure base,” also could function similarly between humans and nature. Perhaps if people repeatedly experience nature as *available* in secure and safe, positive encounters, further proximity-seeking is motivated (which serves to reduce threat), deepening the bond around a secure attachment system (e.g., a cognitive-affective structure; Bowlby, 1988).

The relational attachment process reflects a comprehensive framework for understanding how emotional bonds and relationships develop through patterns of attachment between children and caregivers developed by Ainsworth (1978). Given that interactions with a safe attachment figure is theorized to be incorporated into working models of the self, and since a person tends to assimilate any new bonds, whether with people, or in the case of this present research, with nature, into an existing model, it is feasible to suggest that the human-nature relationship is similarly incorporated into an existing model of the self (Bowlby, 1979). The result is a relational schema that operates automatically and could drastically shape a person's experience in bonding with both nature and others throughout the lifespan.

The cross-sectional study found that secure attachment with nature significantly mediates the relationship between interoceptive awareness and nature connection, thus suggesting that “nature” has the potential to be an attachment figure, serving to facilitate bonding via a secure attachment system and becoming embedded into a relational schema. Given that the cross-sectional study found that human-nature attachment does not covary with human-human attachment, the attachment system between humans and nature is unique, yet built on the same threat attenuation system as human-human bonding.

No research on nature connection has adapted the relational attachment scale to reflect human-nature bonding, and so these findings open up new avenues of exploration. If nature can be a secure attachment figure, and if proximity-seeking to nature is motivated by threat attenuation, and further, if that relationship can be integrated into a working model of the self, a type of human-nature bond is revealed that outpaces the tertiary benefits of connecting to nature, and rather points to a fundamental bond inseparable from models of the self.

This present research thus finds that a core psychological process, one's relational attachment system (Bowlby, 1969) is activated in human-nature bonding. Previous research on place attachment has shown support for the development of *place attachment* through facilitating a sense of connectedness and a positive bond between individuals and places (Nisa et al., 2020). However, secure attachment to nature is different than place attachment; the former based on inter-personal relational processes, the later on the strength of identification with an externalized other.

Interoceptive awareness has also been shown to have a strong link with relational attachment, with suggestive evidence that early developmental attachment teaches the basics of how to know and trust inner bodily signals and sensations just like attachment cues (Oldroyd et al., 2019). As this research suggests, from this foundation of *a coherent bodily self* , relational attachment to nature is more likely. Yet due to the correlational nature of the cross-sectional study, directional causality between these constructs is inconclusive, yet their inter-relatedness is apparent.

### 4.3 A relationship with nature is primarily an emotional bond

A relationship with nature is principally an emotional bond, evidenced by interoceptive awareness (an emotional regulation mechanism) predicting higher levels of nature connectedness, mediated by secure relational attachment (requiring emotional regulation to increase secure attachment). To underscore this finding, out of the eight interoceptive dimensions, emotional awareness and self-regulation were the most highly significantly predictive of nature connectedness. The emotional awareness dimension is defined as the ability to notice how emotions translate as inner bodily signals (e.g., I notice how my body changes when I feel happy/joyful). As stated earlier in the discussion, the capacity to mindfully *notice* these emotions, in addition to self-regulating them, leads to a greater likelihood of secure bonding with nature.

The importance of affect in human-nature bonding is also reflected in nature connection studies. Richardson et al. (2021) and Lumber et al. (2017), building on myriad empirical nature connection studies, claim that nature connectedness is *primarily an emotional bond*, vs. an information or knowledge-based connection. In addition, the Love and Care for Nature scale created by Perkins (2010) seeks to psychometrically capture the construct of love and deep caring for nature as an expression of an explicitly emotional relationship with nature. Used throughout the literature on nature connection, and seen to significantly predict nature connection (Zylstra, 2014), the Love and Care for Nature scale operationalizes this affective domain and supports the importance of emotion in bonding with nature.

This present research underscores this claim, while adding new findings which indicate that awareness of *emotions plus bodily states together* create a mechanism that initiates a spiral of positive emotions stemming from relational security with nature. Therefore, this research parses a difference between merely possessing affective feelings toward nature, and the mechanism of generating those emotions. The results suggest that the human-nature bond is an interpersonal relational process in which a relational schema that impacts one's cognition, affect and behavior is continuously updated vis à vis positive emotional experiences with nature, which motivate further proximity-seeking with nature, resulting in both secure attachment with nature and subsequent behavior that reflects the desire to protect this bond. In sum, ongoing, positive interactions with nature over time will not only increase wellbeing, but motivate further bonding with nature, built on *affective and embodied* interpersonal relational mechanisms.

### 4.4 A relationship with nature is mutually-beneficial

One way the human-nature relationship is mutually beneficial, and even reciprocal, occurs through increasing both wellbeing and flourishing for humans, while simultaneously motivating pro-environmental behaviors on behalf of nature. The cross-sectional study found that nature connectedness significantly predicts wellbeing (measured as eudaimonic happiness), and that those with higher interoceptive awareness and secure attachment to nature were more likely to have increased wellbeing. Wellbeing, measured as eudaimonia (life purpose), is the form of wellbeing most strongly related to nature connection, as indicated in both nature connection empirical studies and a meta-analysis (Nisbet et al., 1998; Pritchard et al., 2020; Shin et al., 2022). Further, this study found that those with higher wellbeing also exhibited significantly higher pro-environmental behaviors. Therefore, this research offers a more precise way to understand a mutually-beneficial relationship.

Mutual benefit can be understood as a gratitude-driven altruism framework (Tam, 2022). The focus on mutual benefit differentiates this present research's definition of nature connection from current nature connection studies, pointing to the importance of *collective altruism* intrinsic to a mutually, reciprocal relationship with nature. Tam (2022) suggests that a gratitude-driven altruism trait might come from the following: it is associated with how frequently the person has contact with nature, how strongly one feels entitled to nature's benefits, and to what extent one perceives nature as humanlike (p. 11).

Reciprocity is currently discussed in decolonisation and Indigenous literature, but is missing from the nature connection studies. Nature connection empirical studies rely on Western notions of “giving back,” including certain behaviors that are determined “pro-environmental” or “pro-nature conservation behavior” (e.g., recycling, planting pollinator-friendly plants, voting for certain policies; Mackay and Schmitt, 2019; Barbett et al., 2020).

While this study measured giving back within the above domains, further research should expand measurement of pro-environmental behaviors to include measures of intersectional environmentalism. Climate justice is intersectionally interconnected with other areas of injustice. In line with this, reciprocal ethics, which can be understood as a gratitude-driven altruism, roots the mutual benefit of the human-nature bond in *relational qualities*. The value of such a mutually-beneficial relationship is reflected in Indigenous scholarship on the importance of the *relational tipping point* vs. single lens focus on the *ecological tipping point*. Whyte (2020) argues that relational qualities like trust, reciprocity and accountability are critical for climate justice, above and beyond individual public or private sphere pro-environmental behaviors.

Such a perspective shifts the focus from research concerned with how to motivate individual pro-environmental behaviors to prevent an *ecological tipping point*, to knowledge and practices that cultivate the relational qualities necessary to prevent *a relational tipping point*. The theoretical proposition is that the human-nature *relationship*, which motivates reciprocal altruism, could be a primary mechanism to mitigate human-driven climate change and human-driven biodiversity loss, via relational qualities, and not just individual behavior. While not mutually exclusive, these paths chart vastly different priorities, epistemologies, ontologies and praxis.

### 4.5 Limitations and further exploration

There are several limitations to this exploratory study. The biomedical science literature indicates that interoceptive awareness is best measured with the heartbeat count or perception task (Garfinkel et al., 2015), not a self-report measure, which was outside the scope of this study. Therefore, future research could seek to replicate these findings by using physiological measures, include a larger sample, and investigate cultural differences. While sample size was sufficient for a correlational study, larger, more diverse, population-level studies would validate and extend these findings and prevent sampling bias.

Since previous research has investigated the efficacy for interoceptive awareness to reduce anxiety, future research could test the efficacy of interoceptive awareness to reduce *climate and eco-anxiety* in comparison to general anxiety disorder and tease apart the inter-relationships. Future research could also parse into the inter-relatedness of interoceptive awareness dimensions and secure attachment, including relational attachment dimensions of approach-avoidance and approach-anxiety. Future work could test if relational attachment predicts nature connection mediated by interoceptive awareness, instead of relational attachment being the mediator, to better understand causality. Theories of body-emotion and self-other bonding could also be brought to bear to better understand the mechanism of body-mind-nature relationships.

Although the sample was drawn from the crowd sourcing platform Prolific Academic, which has their merits compared to Amazon Mechanical Turk (Peer et al., 2017), including transparency about the sample population, options for longitudinal studies, and participant payment rights, they were still mainly derived from Western, Educated, Industrialized, Rich and Democratic nations [WEIRD; (Henrich et al., 2010)]. I sought to correct for this in part by founding this entire research in the perspective of Black, Indigenous and People of Color in the IPA study, but the correlational study did not reflect this population sample diversity. The results of this research are meant to be suggestive and are not causal, nor are they generalizable.

Future research should seek to replicate these results within diverse contexts and to be culturally-specific. In so doing, meaningful intervention design and best-practices can reflect context and culture. In addition, further research could utilize a larger scale structured interview survey study design on a one-on-one or group basis instead of online surveys.

In respect to diversity and equity regarding nature connection research, at least four major strands of research are needed according to Frumkin et al. (2017): (a) patterns of disproportionate exposure; (b) cultural and contextual factors that affect nature preferences and the experience of nature; (c) differing patterns of benefit across different populations; and (d) the possibility that improved access to nature may have unintended negative consequences on vulnerable populations. This research sought to address the cultural and contextual factors that affect the experience of nature, and further research should address the other major strands (Frumkin et al., 2017). There is too little emphasis on culturally and contextually-specific nature connection studies, and much more work is needed to center the perspectives of those whose cultural values already reflect sustainability and kinship with nature, as well as those who are disproportionately affected by climate change. A widespread commitment in nature connection science to diversity, equity and inclusion is essential to protect against perpetuating harm through colonial ideologies.

Regarding interventions to increase nature connectedness, future work could seek to prime interoceptive awareness in a field setting to increase ecological validity. Interventions that pair interoceptive awareness and nature bonding vs. just interoceptive awareness could help to tease apart the role of the body vs. the role of interpersonal bonding in the human-nature relationship. Considering this research points to interpersonal processes of human-nature closeness, future interventions could include primes for animism and anthropomorphism in comparison to just secure relational attachment, to further illuminate how the nature-human bond functions. While this research points to emotion regulation capacities, more work is needed to understand if the mechanism is primarily one of buffering to stressors, in terms of threat-reduction, or is perhaps one of increasing capacity to experience self-transcendent emotions.

In order to examine the collective impacts of interdependent identity shifts occurring in parallel with increased nature connection, future work could seek to understand not just individual responses to an intervention, but how these individuals make up social networks that can reinforce social norms of intersectional environmentalism to one another. Researchers could investigate how inter and intra personal factors fit together with the larger, more fluid and dynamic structural forces that influence environmental justice. To change both individuals and environments requires capturing this reciprocal process. Employing social network analysis would allow researchers to watch the effects of an interoceptive awareness intervention expand and multiply to other members in the network.

Lastly, future research should replicate these findings in partnership with Indigenous scholars using Indigenous research methodologies, including participatory action research and appreciative inquiry, which would increase social and transformational change outcomes (Chilisa, 2020, p. 181).

### 4.6 Conclusion

Human-driven climate change, biodiversity loss and the rapid increase of climate-related disasters indicates that the human-nature relationship is failing. However, this research points to a way to reconnect humans and nature via embodied and secure relational attachment processes. Body awareness creates a foundation for a secure attachment with nature, resulting in positive emotions and behavioral displays of climate care. Thus, the human-nature bond is an emotional regulation strategy, satisfying one's need for attachment security. Body awareness is the starting point for a close relationship with nature: the whole endeavor begins in the senses.

As a lived life process, the body experiences the phenomenal world moment-to-moment, ordering experience and enabling an intimate, felt relationship with the natural world. Given that the human body and the “body” of the Earth are so interconnected, the essentialism of the body points to an ontological rendering of the body as a shared phenomenal reality, and therefore reduces the separatism and individualism undergirding extractive behaviors. Nature and humans are intra-bodied. Humans and nature are kin. Attempts to reduce nature connection to anything less than this fails to account for the immensity of this bond.

This research builds on the literature of the myriad benefits of both nature connection and adult relationships, while addressing the environmental value-action gap through revealing the critical personal factor of interoceptive awareness and highlighting the necessary mechanism of the interpersonal processes occurring in the human-nature relationship. Thus, while advocating for individual increases in nature connection that motivate pro-environmental behaviors is a starting point, it does not go far enough. This research broadens that perspective to include evidence of *an embodied, reciprocal, secure relationship* with nature that could impact communal, structural and societal futures, repealing ideologies that justify domination over nature instead of inter-relatedness with nature. To foster this kind of a relationship with nature, which predicts how committed one is to pro-environmental behaviors in-line with that relationship, requires approaches that center both increasing body awareness and opportunities to interpersonally attach to nature.

With climate collapse and species extinction increasing the urgency of new approaches to address the environmental value-action gap, this research points to the core aspects of body awareness and the closeness of the interpersonal, emotional bond with nature, as *essential* to shifting extractive and damaging human-driven climate change behaviors. The findings of this research should be taken by policy makers, global health practitioners and educators, to design, implement and rapidly scale interventions to increase body awareness in parallel with creating equitable access for the public to experience immersive, emotional encounters with nature. This is a global, low-cost, readily-available solution. Climate change and nature connection interventions should shift from prioritizing exposure to nature or knowledge about nature, to a laser focus on *facilitating the close, emotional, relational development of a bond with nature*. The result could be an interconnected network of human-nature relational fascia, a broadband inoculation to the demands of global imperialism in favor of the values of living in sustainable relational harmony with the Earth. In sum, the strength of the human-nature bond, which is predicated on one's body awareness, is an essential factor in predicting future behavior that either protects or destroys the planet, and so every effort should be made to facilitate it for people everywhere.

## Data availability statement

The raw data supporting the conclusions of this article will be made available by the authors, without undue reservation.

## Ethics statement

The studies involving humans were approved by University of Cambridge Department of Psychology Research Ethics Committee. The studies were conducted in accordance with the local legislation and institutional requirements. The participants provided their written informed consent to participate in this study.

## Author contributions

LB: Conceptualization, Formal analysis, Funding acquisition, Investigation, Methodology, Project administration, Writing – original draft, Writing – review & editing.
